# Photoacoustic Spectroscopy for the Determination of Lung Cancer Biomarkers—A Preliminary Investigation

**DOI:** 10.3390/s17010210

**Published:** 2017-01-21

**Authors:** Yannick Saalberg, Henry Bruhns, Marcus Wolff

**Affiliations:** 1 Hamburg University of Applied Sciences, Heinrich Blasius Institute for Physical Technologies, Berliner Tor 21, 20099 Hamburg, Germany; henry.bruhns@haw-hamburg.de (H.B.); marcus.wolff@haw-hamburg.de (M.W.); 2 University of the West of Scotland, High Street, PA1 2BE Paisley, UK

**Keywords:** photoacoustic spectroscopy, PAS, volatile organic compound, VOC, IR spectra, lung cancer, biomarker, breath analysis, OPO, optical-parametric oscillator

## Abstract

With 1.6 million deaths per year, lung cancer is one of the leading causes of death worldwide. One reason for this high number is the absence of a preventive medical examination method. Many diagnoses occur in a late cancer stage with a low survival rate. An early detection could significantly decrease the mortality. In recent decades, certain substances in human breath have been linked to certain diseases. Different studies show that it is possible to distinguish between lung cancer patients and a healthy control group by analyzing the volatile organic compounds (VOCs) in their breath. We developed a sensor based on photoacoustic spectroscopy for six of the most relevant VOCs linked to lung cancer. As a radiation source, the sensor uses an optical-parametric oscillator (OPO) in a wavelength region from 3.2 µm to 3.5 µm. The limits of detection for a single substance range between 5 ppb and 142 ppb. We also measured high resolution absorption spectra of the biomarkers compared to the data currently available from the National Institute of Standards and Technology (NIST) database, which is the basis of any selective spectroscopic detection. Future lung cancer screening devices could be based on the further development of this sensor.

## 1. Introduction

Over 1.6 million people die due to lung cancer each year [1,2], making this disease one of the leading causes of death worldwide. So far, no methodical general screening for lung cancer has been established [3,4]. A preventive medical examination could save thousands of lives per year, as the survival rates decrease rapidly with increasing stages (47% in Stage 1, 1%–2% in Stage 4) [5]. An early diagnosis could also considerably relieve the health care system by reducing the cost of cancer treatment. One promising approach for future lung cancer screening could be breath tests based on the analysis of expiratory air [6,7]. It would make use of the fact that lung cancer patients exhale certain substances in different combinations and concentrations as compared to healthy persons [8,9,10]. These substances belong to the group of volatile organic compounds (VOCs) and could be used as biomarkers in the future, according to the definition of the WHO [11]. These VOCs are either generated directly in the lungs or transported there via bloodstream from other parts of the body. Gas exchange in the lungs, in combination with the high volatility of the substances, leads to exhalation and allows for VOC detection in the breath. Several authors claim that breath tests with high sensitivity and significance are possible [8,9,10]. However, authors do not agree on which VOCs are the most relevant. This is the main reason that, up to now, no lung cancer breath test has been established, although some VOCs have been linked to this disease for decades. Since the literature refers to these VOC substances as biomarkers, we will utilize this term in this paper as well.

A literature review performed in 2016 [12] revealed the six most frequently listed and, therefore, most relevant lung cancer biomarkers found in breath. The review did not distinguish between different stages or subtypes of lung cancer, meaning that these VOCs could provide a suitable basis for future screening examinations, although some of them have been linked to other diseases as well.

We have developed a photoacoustic sensor that enables sensitive detection of the six most relevant of these vaporous compounds. It is based on a continuous-wave optical-parametric oscillator (OPO) whose spectrally narrow emission is widely tunable in the mid-infrared region between 3.2 µm and 3.5 µm. This part of the spectrum is particularly well-suited for spectroscopic detection because it relates to the C–H stretching vibration, and hydrocarbons show very strong and characteristic absorption here [13,14]. Furthermore, atmospheric components, such as N_2_, O_2_, H_2_O, Ar, or CO_2,_ show no or only very weak absorption which minimizes possible cross sensitivities. 

## 2. Materials and Methods

### 2.1. Experimental Setup

Photoacoustic spectroscopy (PAS) takes advantage of the photoacoustic effect described by A. G. Bell in 1881 [15]. The effect is based on the absorption of electromagnetic radiation and the transfer of this absorbed energy into kinetic energy through molecular collisions. This non-radiative relaxation is equivalent with a temperature increase of the irradiated volume. A modulated irradiation generates a temperature variation that coincides with a pressure modulation. This sound wave can be easily detected by a microphone. The advantage of PAS over traditional transmission spectroscopy is that the signal is directly proportional to the absorption coefficient of the substance. As long as the absorption is not saturated, the signal is proportional to the substance concentration as well. PAS is hence considered an offset-free technique. Another advantage is based on acoustic resonances of the sample containing chamber. Modulating the radiation source with a frequency that is equivalent to an acoustic mode of this sample cell leads to an amplification of the generated signal and, therefore, enhances the signal-to-noise ratio (SNR) [16,17]. In comparison to mass spectrometry and gas chromatography, PAS has the advantage that no sample preparation is required, and the sample is not destroyed during the measurement [18].

**Figure 1:**
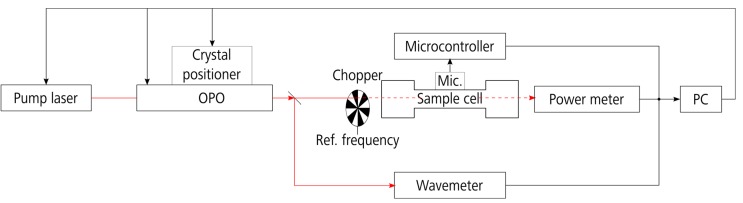
Experimental setup of the photoacoustic sensor.

Figure 1 shows the experimental setup of the photoacoustic sensor. We used a continuous-wave optical-parametric oscillator (cw-OPO) as radiation source (Argos Model 2400-BB-5 Module C, Lockheed Martin Aculight, Bothel, WA, USA). The OPO is equipped with a modified fiber pump laser in order to get a narrower emission linewidth of less than 500 pm full width at half maximum. The idler wavelength can be spectrally tuned between 3.2 µm and 3.5 µm with a maximum emission power of approximately 2 W. Spectral tuning is achieved by adjusting the position of the fan-poled optical nonlinear crystal (coarse tuning) and the angle of an etalon (fine tuning). Both parameters are controlled via USB interface and MATLAB control software. Modulation of the OPO emission is achieved using a mechanical chopper (300CD, Scitec Instruments, Redruth, UK). Details of the OPO principle and its control are described in an earlier publication [19]. 

Measurements were taken with the test gases filled into the H-shape sample cell that was designed to have its first longitudinal resonance frequency at 2.7 kHz [20]. The cell is made of aluminum and sealed on both ends with calcium fluoride (CaF_2_) windows. The flexible hoses leading the gas sample into the cell are made of polytetrafluoroethylene (PTFE—Teflon). These connections are resistant against many chemicals and substances show low adhesion. In order to measure the OPO wavelength a fraction of the laser beam (7%) is separated with a beam splitter and guided to a laser spectrum analyzer (721A-IR, Bristol Instruments, Victor, NY, USA) featuring a precision of up to ±0.2 ppm of the absolute wavelength. The optical emission power of the OPO was measured with a power meter behind the cell (Thermal head model 3A-FS-SH, Ophir Optronics, Jerusalem, Israel). The loss in laser power due to absorption can be neglected for the measured VOCs. According to reference spectra from Pacific Northwest National Laboratory (PNNL), the compound with the strongest absorption (ethylbenzene) shows a maximum absorbance of 400 × 10 ^−6^ at a concentration of 1 ppm, a path length of 1 m and a temperature of 25 °C [21]. This corresponds to a maximum power loss of less than 1.2% behind our sample cell filled with 100 ppm test gas. The power meter, however, provides a precision of only ±3% according to the datasheet. The measured emission power behind the cell is used to normalize the photoacoustic (PA) signal.

The PA signal was detected using a microelectromechanical systems (MEMS) microphone (INMP441 from InvenSense, San Jose, CA, USA). The microphone possesses a high sensitivity of −25 dBFS and a signal-to-noise ratio of 61 dBA. It integrates a digital I^2^S interface securing noise-free data transmission. The I^2^S data of the microphone was further processed using a microcontroller (PIC32 from Microchip). The acoustic signal was recorded with a frequency of 7.8 kHz, thus fulfilling the sampling theorem. The amplitude of the acoustic input signal at the resonance frequency was calculated using the Goertzel algorithm implemented in C language on the PIC32 [22,23,24,25]. The Goertzel algorithm is a digital filter that calculates one specific frequency bin of the discrete Fourier transform (DFT). Further details on the MEMS microphone and the data processing are described in earlier publications [25,26].

### 2.2. Lung Cancer Biomarkers

In 2016, a systematic literature research was performed to identify compounds that could serve as lung cancer biomarkers for future breath tests [12]. The most frequently listed substances are 2-butanone [18,27,28,29,30], 1-propanol [18,27,29,30,31], isoprene [18,31,32,33], ethylbenzene [27,33,34,35], styrene [32,33,35,36], and hexanal [9,32,34,37]. All substances have been linked to lung cancer but some not exclusively, and the pathways are still under investigation. Some of these compounds might also be of exogenous origin [9,38]. Table 1 lists the most relevant substances in order of priority [12]. The priorities are based on the number of research groups that declared a certain VOC a biomarker for lung cancer.

Measurements were conducted on the six VOCs of Priorities 1 and 2. They were acquired from Sigma-Aldrich in liquid state (at room temperature). Gaseous mixtures with a VOC concentration of 100 ppm in nitrogen were prepared in Tedlar bags. These nitrogen (purity: 5.0) filled polyvinyl fluoride (PVF) bags represent the most commonly used mix and storage containers for gas sampling in the field of breath analysis [39,40]. These containers do not completely prevent adsorption and diffusion through the bag. However, due to our short storage time of approximately one hour, these effects can be neglected [41,42].

The liquid VOC was drawn up with a 2 µL syringe and injected into the 1 L Tedlar bag. The small puncture hole in the bag was subsequently sealed with tape. The required liquid volume $V_{VOC}$ for a concentration of 100 ppm can be calculated by using the following equation:

(1) $$V_{VOC}=\frac{MV_{mix}c}{\delta V_{m}}$$

with M representing the VOC’s molar mass, $V_{mix}$ the volume of the Tedlar bag (1 L), c the desired concentration of VOC (100 ppm), δ the density of the liquid VOC, and $V_{m}$ the molar volume of an ideal gas at room temperature and ambient pressure. During the preparation of the sample, the measuring cell was evacuated for one hour in order to remove any traces from previous measurements. The used chemistry-hybrid pump is a combination of a two-stage rotary vane pump and a two-stage chemistry diaphragm pump (RC 6 from Vacuubrand). 

Since the evaporation rates of VOCs are high compared to water, it can be assumed that the VOC droplet is completely evaporated after a storage time of one hour [43]. Thereupon, the low pressure of the evacuated system was used to suck the gas mixture from the bag into the sample cell, resulting in ambient pressure inside the cell (approximately 1024 hPa). The screw cap valve of the Tedlar bag was used for the connection to ensure sealing. This procedure for the generation of VOC gas mixtures is established among researchers in this field [44]. After the gas transfer from the preparation bag to the sample cell, the gas in- and outlet of the cell were closed. Both the transfer system and the sample cell were at room temperature. Because of the comparably small sample cell volume (30 mL) and minimal temperature differences between gas and cell, we assume to have reached a thermal equilibrium in less than a minute. The measurement process was started three minutes after insertion of the gas.

### 2.3. Measurements

We measured photoacoustic spectra of the six most relevant lung cancer biomarkers using the experimental setup described above. All measurements were performed under static conditions with no gas flow. In order to do so, the spectral emission of the OPO was tuned from 3.2 µm to 3.5 µm controlling the crystal position and the etalon angle. However, the spectral tuning is not continuous due to the phase-matching condition. Only discrete wavelength values are accessible. Since the wavelength steps are unequally spaced, the spectral resolution cannot be expressed by a single value.

**Figure 2:**
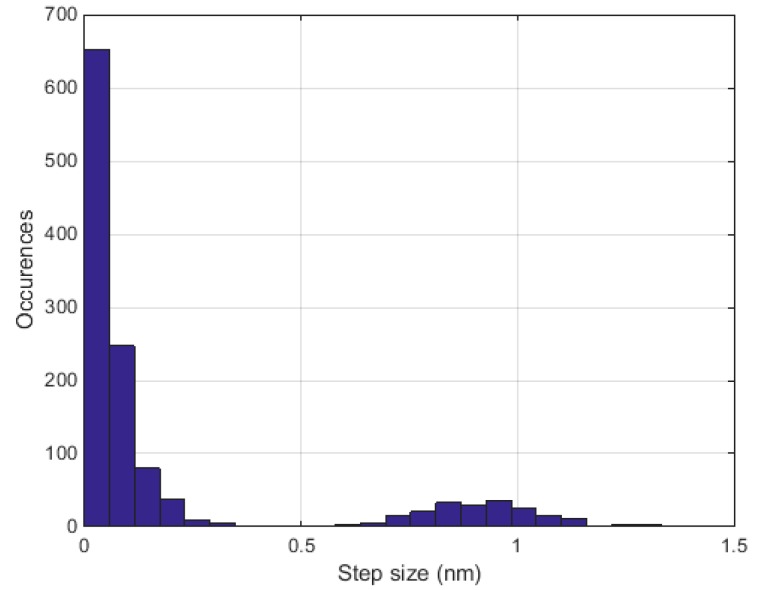
Number of occurrences of optical–parametric oscillator wavelength step sizes between 3.2 µm and 3.5 µm.

Figure 2 shows the number of occurrences of wavelength step sizes between 3.2 µm and 3.5 µm. The displayed data originates from ethylbenzene, but the other VOCs do not deviate significantly. The step size between accessible wavelengths has a mean value of 0.20 nm. The relatively large standard deviation of 0.34 nm is owed to the second distribution, with step sizes between 0.7 nm and 1.2 nm and considerably fewer occurrences. The corresponding spectral resolution, however, is sufficient to measure VOC spectra considering the fact that these are comparably large molecules and that their absorption features correspondingly broad. Furthermore, the spectral resolution is better than that of many Fourier transform infrared spectrometers (FTIR). At each OPO configuration, i.e., at each specific emission wavelength, we performed ten measurements and averaged the results.

**Figure 3:**
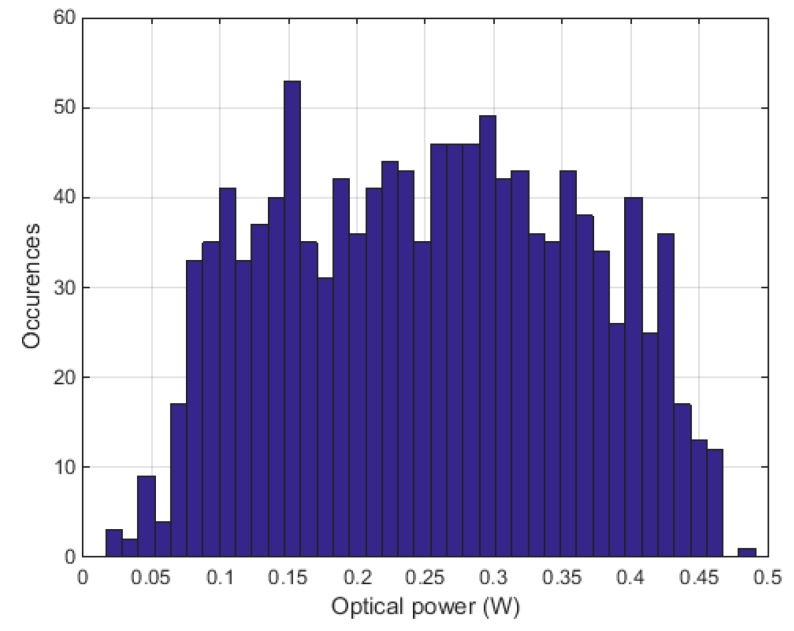
Number of occurrences of the optical–parametric oscillator output power between 3.2 µm and 3.5 µm.

The OPO’s emission power strongly depends on the emission wavelength, i.e., on the crystal position and the etalon angle. It can change drastically from one wavelength configuration to the next. In order to eliminate the dependency of the photoacoustic signal on the optical output power of the OPO, the microphone signal was normalized in regard to the output power. Figure 3 shows the number of occurrences of the OPO output power between 3.2 µm and 3.5 µm for the ethylbenzene measurement. The average power is equal to 0.25 W, whereas the standard deviation is 0.11 W. Again, the other VOCs do not deviate significantly. 

In order to determine the noise level, we performed an additional measurement with a nitrogen-filled cell (purity: 5.0). At each of the five OPO wavelengths, evenly distributed over the spectral region, we measured 300 values of the photoacoustic signal. As with the spectra measurements, these values were grouped into blocks of ten and averaged. Of these 300 × 5/10 = 150 measurements, we calculated the mean value and the standard deviation.

## 3. Results

**Figure 4:**
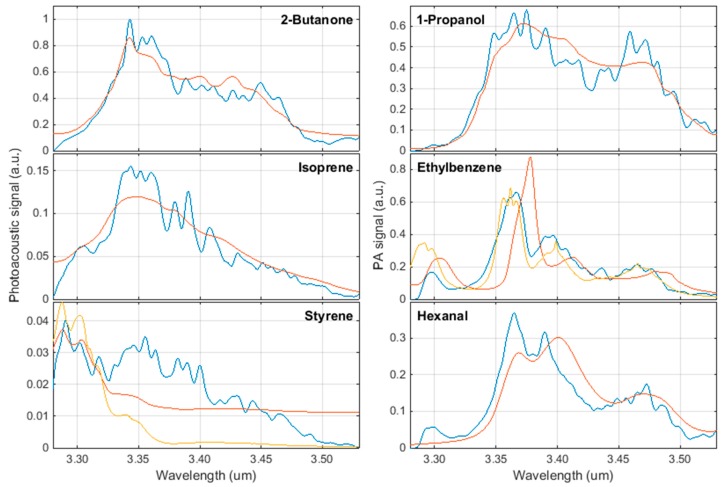
Biomarker spectra (**blue**: Measurement; **red**: NIST; **yellow**: PNNL) measured at a concentration of 100 ppm in nitrogen at atmospheric conditions (294 K, 1024 hPa).

Figure 4 shows the photoacoustic spectra of the six most relevant lung cancer biomarkers listed in Table 1. The data is available as supplementary data from the journal. Each diagram also includes the absorption spectrum from the National Institute of Standards and Technology (NIST). The diagrams of ethylbenzene and styrene additionally display PNNL data. Since NIST does not provide any parameters of the measurements (such as concentration or path length), the spectra are purely qualitative. In order to enable comparability, the NIST spectra are scaled to the same root-mean-square, which represents the area under the curve, as the measured photoacoustic spectra. 

Detection limits of the photoacoustic sensor were estimated for each biomarker. While the mean value of noise can be considered a subtractable offset due to absorption in the windows etc., the standard deviation determines the detection limit. The maximum signal in each spectrum must be larger than this fluctuating noise. This allows calculation of signal-to-noise ratios and idealized limits of detection (LODs), representing the theoretically lowest measureable concentration for each biomarker. The transformation from $SNR_{Amp}$ (amplitude) to a decibel level was achieved using $SNR_{dB}=20log_{10}\left(SNR_{Amp}\right)$. Table 2 lists the results.

## 4. Discussion

The photoacoustic sensor based on a cw-OPO is able to measure the six most relevant biomarkers for lung cancer, namely, 2-butanone, 1-propanol, isoprene, ethylbenzene, styrene, and hexanal, with high detection sensitivity.

Three of the six photoacoustic spectra in the wavelength range between 3.2 µm and 3.5 µm are in good agreement with NIST spectra. The measured ethylbenzene spectrum, however, shows a wavelength shift of approximately 20 nm towards lower wavelengths. The fact that the measurement is in good accordance with PNNL data supports PNNL with regard to this compound. The photoacoustic hexanal spectrum is similarly shifted compared to its NIST counterpart. Unfortunately, no PNNL data is available for this substance. The spectral deviation could be a result of the fact that NIST spectra are measured with broadband FTIR spectrometers, which provide a wide wavelength range at the costs of wavelength accuracy. Since the future biomarker sensor will be operating on the basis of measured reference spectra, the deviation to the database has no implications on the further development. The difference in the spectrum of styrene cannot be put into perspective. Both reference spectra deviate considerably from the measurement. We will investigate this further.

Each lung cancer biomarker shows a very characteristic spectrum in the mid-infrared region. The spectral resolution of the photoacoustic sensor can be expressed by a spectral emission linewidth of the OPO of less than 0.5 nm and an average tuning step size of 0.20 nm. Therefore, the measured photoacoustic spectra provide a considerably higher resolution than the spectroscopic data currently provided by NIST and PNNL. This will enable identification of compounds by their characteristic absorption peaks. The idealized noise equivalent detection limits of the sensor for a single VOC range between 5 ppb and 142 ppb. Some of the VOCs feature a comparably large dipole moment and can therefore be considered chemically sticky. The consequence of the according adsorption to the cell walls would be a reduction of the reference VOC concentration inside the cell. This is not taken into account for the determination of the LOD. Therefore, the resulting values represent more upper limits of the true LOD.

As a next step, measurements at different VOC concentrations will be conducted in order to verify its sensitivity and to determine the true detection limits. This will be followed by measurements of biomarker mixtures. Spectra will be analyzed using a sophisticated evaluation algorithm. Potential techniques include the multivariate analysis, principle component analysis (PCA), neuronal network approaches, and fuzzy logic [45]. The most significant wavelengths for the analysis will be determined using interrelation miner or the like [46]. The data at hand, i.e., the high-resolution absorption spectra, will enable the identification of single compounds and, thus, high detection selectivity. The photoacoustic analyzer will have the potential to serve as a basis for the development of a lung cancer screening device. A comparison with existing technologies in the detection of VOCs as well as a discussion of pros and cons of the new analyzer will be performed once the processing algorithm is finished.

## Figures and Tables

**Table 1:** Most relevant biomarkers [12].

Priority	CAS	Names
1	78-93-3	2-butanone, methyl ethyl ketone
	71-23-8	1-propanol, n-propanol
2	78-79-5	isoprene, 2-methyl-1,3-butadiene
	100-41-4	ethylbenzene
	100-42-5	styrene, ethenylbenzene
	66-25-1	hexanal
3	67-64-1	acetone, propanone
	107-87-9	2-pentanone, methyl propyl ketone
	67-63-0	2-propanol, isopropylalcohol, isopropanol
	124-18-5	decane
	71-43-2	benzene
	111-71-7	heptanal
	106-97-8	butane
	123-38-6	propanal
	109-66-0	n-pentane

**Table 2:** Detection limits for lung cancer biomarkers.

Name	SNR (100 ppm)	LOD (ppb)
2-butanone	17,629 => 84.92 dB	5.7
1-propanol	11,969 => 81.56 dB	8.4
isoprene	2,729 => 68.72 dB	36.6
ethylbenzene	11,645 => 81.32 dB	8.6
styrene	706 => 56.98 dB	141.6
hexanal	6491 => 76.25 dB	15.4

## References

[1] Ferlay J., Soerjomataram I., Dikshit R., Eser S., Mathers C., Rebelo M., Parkin D.M., Forman D., Bray F. (2015). Cancer incidence and mortality worldwide: sources, methods and major patterns in GLOBOCAN 2012. Int. J. Cancer.

[2] Stewart B.W., Wild C.P. (2014). World Cancer Report 2014.

[3] Smith R.A., Manassaram-Baptiste D., Brooks D., Cokkinides V., Doroshenk M., Saslow D., Wender R.C., Brawley O.W. (2014). Cancer screening in the United States, 2014: A review of current American Cancer Society guidelines and current issues in cancer screening. CA Cancer J. Clin..

[4] Moyer V.A. (2014). Screening for lung cancer: U.S. Preventive Services Task Force recommendation statement. Ann. Intern. Med..

[5] Rami-Porta R., Crowley J.J., Goldstraw P. (2009). The revised TNM staging system for lung cancer. Ann. Thorac. Cardiovasc. Surg..

[6] Taivans I., Bukovskis M., Strazda G., Jurka N. (2014). Breath testing as a method for detecting lung cancer. Expert Rev. Anticancer Ther..

[7] Van’t Westeinde S.C., van Klaveren R.J. (2011). Screening and early detection of lung cancer. Cancer J..

[8] Ligor T., Pater Ł., Buszewski B. (2015). Application of an artificial neural network model for selection of potential lung cancer biomarkers. J. Breath Res..

[9] Poli D., Goldoni M., Corradi M., Acampa O., Carbognani P., Internullo E., Casalini A., Mutti A. (2010). Determination of aldehydes in exhaled breath of patients with lung cancer by means of on-fiber-derivatisation SPME-GC/MS. J. Chrom. B..

[10] Phillips M., Cataneo R.N., Cummin A.R.C., Gagliardi A.J., Gleeson K., Greenberg J., Maxfield R.A., Rom W.N. (2003). Detection of lung cancer with volatile markers in the breath. Chest.

[11] WHO International Programme on Chemical Safety Biomarkers in Risk Assessment: Validity and Validation. http://www.inchem.org/documents/ehc/ehc/ehc222.htm.

[12] Saalberg Y., Wolff M. (2016). VOC breath biomarkers in lung cancer. Clin. Chim. Acta.

[13] Tittel F.K., Richter D., Fried A., Sorokina I.T., Vodopyanov K.L. (2003). Mid-Infrared Laser Applications in Spectroscopy. Solid-State Mid-Infrared Laser Sources.

[14] Klingbeil A.E. (2007). Mid-IR Laser Absorption Diagnostics for Hydrocarbon Vapor Sensing in Harsh Environments. Ph.D Thesis.

[15] Bell A.G. (1881). Upon the Production of Sound by Radiant Energy. Philos. Mag..

[16] Michaelian K.H. (2010). Photoacoustic IR Spectroscopy: Instrumentation, Applications and Data Analysis.

[17] Sigrist M.W. (1994). Laser photoacoustic spectrometry for trace gas monitoring. Analyst.

[18] Bajtarevic A., Ager C., Pienz M., Klieber M., Schwarz K., Ligor M., Ligor T., Filipiak W., Denz H., Fiegl M. (2009). Noninvasive detection of lung cancer by analysis of exhaled breath. BMC Cancer.

[19] Bruhns H., Saalberg Y., Wolff M. (2015). Photoacoustic Hydrocarbon Spectroscopy Using a Mach-Zehnder Modulated cw OPO. Sens. Transducers.

[20] Elia A., Lugarà P.M., Di Franco C., Spagnolo V. (2009). Photoacoustic techniques for trace gas sensing based on semiconductor laser sources. Sensors.

[21] Pacific Northwest National Laboratory. https://secure2.pnl.gov/nsd/nsd.nsf/Welcome.

[22] Goertzel G. (1958). An Algorithm for the Evaluation of Finite Trigonometric Series. Am. Math. Monthly.

[23] Oppenheim A.V., Schafer R.W. (2010). Discrete-Time Signal Processing.

[24] Sysel P., Rajmic P. (2012). Goertzel algorithm generalized to non-integer multiples of fundamental frequency. J. Adv. Signal Process..

[25] Bruhns H., Marianovich A., Wolff M. (2014). Photoacoustic Spectroscopy Using a MEMS Microphone with Inter-IC Sound Digital Output. Int. J. Thermophys..

[26] Bruhns H., Marianovich A., Rhein S., Wolff M. Digital MEMS Microphone with Inter-IC Sound Interface for Photoacoustic Spectroscopy. Proceedings of the 14th International Meeting on Chemical Sensors, IMCS 2012.

[27] Buszewski B., Ligor T., Jezierski T., Wenda-Piesik A., Walczak M., Rudnicka J. (2012). Identification of volatile lung cancer markers by gas chromatography-mass spectrometry: Comparison with discrimination by canines. Anal. Bioanal. Chem..

[28] Fu X.-A., Li M., Knipp R.J., Nantz M.H., Bousamra M. (2014). Noninvasive detection of lung cancer using exhaled breath. Cancer Med..

[29] Gordon S.M., Szidon J.P., Krotoszynski B.K., Gibbons R.D., O’Neill H.J. (1985). Volatile organic compounds in exhaled air from patients with lung cancer. Clin. Chem..

[30] Ligor M., Ligor T., Bajtarevic A., Ager C., Pienz M., Klieber M., Denz H., Fiegl M., Hilbe W., Weiss W. (2009). Determination of volatile organic compounds in exhaled breath of patients with lung cancer using solid phase microextraction and gas chromatography mass spectrometry. Clin. Chem. Lab. Med..

[31] Ulanowska A., Kowalkowski T., Trawińska E., Buszewski B. (2011). The application of statistical methods using VOCs to identify patients with lung cancer. J. Breath Res..

[32] Chen X., Xu F., Wang Y., Pan Y., Lu D., Wang P., Ying K., Chen E., Zhang W. (2007). A study of the volatile organic compounds exhaled by lung cancer cells in vitro for breath diagnosis. Cancer.

[33] Poli D., Carbognani P., Corradi M., Goldoni M., Acampa O., Balbi B., Bianchi L., Rusca M., Mutti A. (2005). Exhaled volatile organic compounds in patients with non-small cell lung cancer: cross sectional and nested short-term follow-up study. Respir. Res..

[34] Handa H., Usuba A., Maddula S., Baumbach J.I., Mineshita M., Miyazawa T. (2014). Exhaled breath analysis for lung cancer detection using ion mobility spectrometry. PLoS One.

[35] Rudnicka J., Kowalkowski T., Ligor T., Buszewski B. (2011). Determination of volatile organic compounds as biomarkers of lung cancer by SPME-GC-TOF/MS and chemometrics. J. Chromatogr. B.

[36] Phillips M., Gleeson K., Hughes J.M.B., Greenberg J., Cataneo R.N.,  Baker L., McVay W.P. (1999). Volatile organic compounds in breath as markers of lung cancer: A cross-sectional study. Lancet.

[37] Fuchs P., Loeseken C., Schubert J.K., Miekisch W. (2010). Breath gas aldehydes as biomarkers of lung cancer. Int. J. Cancer.

[38] Szulejko J.E., McCulloch M., Jackson J., McKee D.L., Walker J.C., Solouki T. (2010). Evidence for Cancer Biomarkers in Exhaled Breath. IEEE Sensors J..

[39] Krilaviciute A., Heiss J.A., Leja M., Kupcinskas J., Haick H., Brenner H. (2015). Detection of cancer through exhaled breath: a systematic review. Oncotarget.

[40] Mochalski P., King J., Unterkofler K., Amann A. (2013). Stability of selected volatile breath constituents in Tedlar, Kynar and Flexfilm sampling bags. Analyst.

[41] Beauchamp J., Herbig J., Gutmann R., Hansel A. (2008). On the use of Tedlar® bags for breath-gas sampling and analysis. J. Breath Res..

[42] Steeghs M.M.L., Cristescu S.M., Harren F.J.M (2007). The suitability of Tedlar bags for breath sampling in medical diagnostic research. Physiol. Meas..

[43] Heymes F., Aprin L., Bony A., Forestier S., Cirocchi S., Dusserre G. (2013). An experimental investigation of evaporation rates for different volatile organic compounds. Proc. Safety Prog..

[44] Hirschmann C.B., Koivikko N.S., Raittila J., Tenhunen J., Ojala S., Rahkamaa-Tolonen K., Marbach R., Hirschmann S., Keiski R.L. (2011). FT-IR-cPAS--new photoacoustic measurement technique for analysis of hot gases: a case study on VOCs. Sensors.

[45] Kessler W. (2007). Multivariate Datenanalyse: Für die Pharma-, Bio- und Prozessanalytik.

[46] Kohl I., Beauchamp J., Cakar-Beck F., Herbig J., Dunkl J., Tietje O., Tiefenthaler M., Boesmueller C., Wisthaler A., Breitenlechner M. (2013). First observation of a potential non-invasive breath gas biomarker for kidney function. J. Breath Res..

